# Development and Evaluation of a Modified Fixed-Dose Combination Antihypertensive Tablet Containing S-Amlodipine Besylate: A Bioequivalence and Stability Study

**DOI:** 10.3390/pharmaceutics17091235

**Published:** 2025-09-22

**Authors:** Hyeon Woo Moon, Jin-Hyuk Jeong, Chun-Woong Park

**Affiliations:** 1 College of Pharmacy, Chungbuk National University, Cheongju 28160, Republic of Korea; mhw2@dkpharm.co.kr (H.W.M.); jinddong92@gmail.com (J.-H.J.); 2 Dongkook Pharmaceutical Co., Ltd., Suwon 16229, Republic of Korea

**Keywords:** S-amlodipine besylate, olmesartan medoxomil, hydrochlorothiazide, fixed-dose combination, bilayer tablet, stability, bioequivalence, hypertension, antihypertensive therapy

## Abstract

**Background**/**Objectives**: Fixed-dose combination (FDC) antihypertensive medications containing olmesartan medoxomil, amlodipine besylate, and hydrochlorothiazide are widely used for the treatment of essential hypertension. Although effective, the use of racemic amlodipine, which contains both active S(−)-amlodipine and inactive R(+)-amlodipine, has been associated with dose-dependent adverse effects, such as peripheral edema. S-amlodipine, a pharmacologically active enantiomer, provides comparable antihypertensive efficacy at half the dose with a lower incidence of side effects. **Methods**: In this study, a modified FDC formulation was developed by replacing racemic amlodipine with S-amlodipine to enhance tolerability while maintaining therapeutic efficacy. **Results**: A bilayer tablet design was employed to minimize the formation of impurities and ensure formulation stability, which was confirmed under stress and accelerated conditions. In vitro dissolution testing demonstrated pharmaceutical equivalence with the marketed reference FDC, and an in vivo pharmacokinetic study confirmed bioequivalence. **Conclusions**: These results suggest that the newly developed S-amlodipine besylate-containing FDC tablet is a viable alternative to existing olmesartan/amlodipine/hydrochlorothiazide combinations, offering comparable efficacy and pharmacokinetic properties with the potential for improved safety and patient adherence in the management of hypertension.

## 1. Introduction

Olmesartan medoxomil is an angiotensin II receptor blocker (ARB) used for the treatment of hypertension in adults and pediatric patients aged 6 years and older [1]. As a prodrug, it is rapidly hydrolyzed in the gastrointestinal tract to its pharmacologically active metabolite, olmesartan [2]. Olmesartan selectively inhibits the binding of angiotensin II to the AT1 receptor in the vascular smooth muscle, thereby preventing vasoconstriction and reducing blood pressure [3]. Clinical studies have demonstrated that olmesartan medoxomil provides significant and sustained antihypertensive effects, with a favorable safety profile [4]. Its pharmacokinetic properties include a relatively long half-life and support for once-daily dosing, which may enhance patient adherence to therapy [5].

Hydrochlorothiazide is a thiazide diuretic widely used for treating hypertension and edema [6]. It works by inhibiting the chloride–sodium cotransporter in the distal convoluted tubules of the kidneys and increasing the excretion of sodium and water, which reduces blood volume and lowers blood pressure [7]. Hydrochlorothiazide is approved by the U.S. Food and Drug Administration (FDA) for the treatment of essential hypertension and the management of peripheral edema associated with conditions such as congestive heart failure, cirrhosis, nephrotic syndrome, and edema related to corticosteroid or estrogen therapy [8,9]. In addition, it has been used off-label for conditions such as nephrogenic diabetes insipidus and for preventing calcium-containing kidney stones [10]. The long-established efficacy and safety profile of hydrochlorothiazide has cemented its role as the cornerstone of antihypertensive therapy [11,12].

Amlodipine besylate, a dihydropyridine calcium channel blocker, is widely used for its antihypertensive and antiangiogenic effects [13]. It effectively lowers blood pressure by inhibiting calcium influx into the vascular smooth muscle and inducing vasodilation [14]. Amlodipine besylate is clinically used to treat hypertension, chronic stable angina, and vasospastic angina [15,16]. It supports once-daily dosing and improves patient adherence due to its long half-life [17]. Studies have demonstrated that amlodipine besylate effectively reduces the frequency of angina episodes and improves exercise tolerance in patients with coronary artery disease [18]. Amlodipine is typically administered as a racemic mixture containing equal amounts of the R(+) and S(−) enantiomers. Notably, the S(−) enantiomer, S-amlodipine, selectively inhibits L-type calcium channels, induces vasodilation, and effectively lowers blood pressure, thus exhibiting significant pharmacological activity. In contrast, R(+) enantiomers are considered pharmacologically inactive [19]. Clinical studies suggest that administering S-amlodipine at half the dose of racemic amlodipine may achieve an antihypertensive efficacy comparable to that of racemic amlodipine with an improved safety profile. For example, in a randomized, double-blind clinical trial of mild-to-moderately hypertensive patients over 8 weeks, S-amlodipine nicotinate (2.5 mg) demonstrated similar diastolic blood pressure-lowering effects to 5 mg of amlodipine besylate, with comparable tolerability [20]. Several clinical studies directly comparing S-amlodipine and racemic amlodipine have consistently reported that S-amlodipine achieves equivalent blood pressure reduction with a markedly lower incidence of adverse events, particularly peripheral edema, which is often attributed to the inactive R(+)-enantiomer. This superior safety profile has been observed in both short-term randomized trials and long-term follow-up studies, further supporting the clinical relevance of chiral switching from racemic to enantiopure formulations [21,22]. S-amlodipine offers a targeted approach by delivering an active enantiomer and improving therapeutic outcomes while minimizing side effects [22]. This enantiomer-specific strategy aligns with the broader trend in drug development toward optimizing drug efficacy and safety through chiral switching [23].

Hypertension management has evolved with the recognition that optimal blood pressure control often requires multiple antihypertensive agents [24]. Fixed-dose combination (FDC) drugs, which combine two or more antihypertensive medications into a single tablet, are essential for the treatment of hypertension. The use of FDCs is based on the fact that several hypertensive patients require more than one drug to achieve adequate blood pressure control [25]. FDCs offer several advantages, including improved patient adherence, simplified treatment regimens, and potential for better blood pressure management. FDCs address common challenges in hypertension management, such as poor adherence to complex multidrug regimens and the risk of suboptimal blood pressure reduction. By combining drugs with complementary mechanisms of action, FDCs provide a more comprehensive approach for blood pressure control, potentially improving treatment outcomes and reducing the burden of cardiovascular diseases [26]. Clinical evidence has demonstrated that FDCs are not only as effective as, but often superior to, individual components in terms of blood pressure reduction, side effects, and patient satisfaction [27]. Consequently, FDCs have become increasingly prevalent in clinical practice, offering an integrated approach to manage hypertension while meeting the growing demand for simplified, patient-friendly treatment options.

Sevikar HCT^®^ is a FDC medication designed for the treatment of hypertension, combining three active pharmaceutical ingredients (APIs): olmesartan medoxomil, amlodipine besylate, and hydrochlorothiazide [28]. Each component targets a distinct pathway involved in blood pressure regulation. The combination of these three APIs in Sevikar HCT^®^ provides a comprehensive approach for hypertension management. FDCs such as Sevikar HCT^®^ have become increasingly significant in clinical practice due to their ability to simplify treatment regimens, improve patient adherence, and effectively address the multifactorial nature of hypertension. FDCs integrate complementary mechanisms of action to offer enhanced blood pressure control, which is particularly beneficial in patients with an inadequate response to monotherapy or those requiring multiple medications to achieve optimal blood pressure targets. Clinical evidence has demonstrated that Sevikar HCT^®^ is both safe and effective in lowering blood pressure in patients with essential hypertension, providing sustained antihypertensive effects with a once-daily dosing regimen that enhances patient compliance [29]. Thus, Sevikar HCT^®^ represents an important therapeutic for the integrated management of hypertension and reduces cardiovascular risk and improves patient outcomes [30].

The FDC therapy has gained significant importance in modern pharmacotherapy owing to its potential to enhance synergistic efficacy, simplify dosing regimens, and reduce side effects. FDCs can improve patient compliance and therapeutic outcomes, especially in chronic diseases that require polypharmacy, such as hypertension, diabetes, and HIV, by combining multiple active pharmaceutical ingredients (APIs) into a single dosage form. However, the development of solid oral dosage forms containing multiple APIs presents notable challenges, primarily related to the chemical and physical incompatibilities between APIs or between APIs and excipients. This incompatibility may cause drug degradation, reduced therapeutic efficacy, or safety concerns. To overcome these challenges, various formulation strategies have been developed to physically separate incompatible components into a single dose. Among these, multilayer tablet technology has emerged as a promising platform that allows the co-formulation of multiple APIs with different release profiles or compatibility characteristics. Multilayer tablets exert synergistic effects, display dual-release kinetics, and mitigate physicochemical incompatibility by segregating APIs into discrete layers. Recent advances in multilayer compression equipment and formulation techniques have enhanced the commercial feasibility of these systems despite inherent challenges such as layer separation, process complexity, and stringent quality control requirements [31]. In addition to multilayer tablets, innovative formulation approaches, such as API coatings, have been extensively explored. Fluidized bed-coating devices, such as the Wurster apparatus, enable the application of polymer films around individual drug particles, effectively isolating sensitive APIs from direct interactions. This strategy not only improves stability but also enhances critical manufacturing attributes, including powder flowability and compressibility [32]. Another notable approach is the polypill or polycap formulation, which encapsulates multiple drugs within a single capsule, thereby improving patient adherence and simplifying therapeutic regimens [33].

We developed a modified FDC antihypertensive formulation by replacing amlodipine with its pharmacologically active enantiomer, S-amlodipine, in the commercially available Sevikar HCT^®^, to improve drug stability and therapeutic performance through multilayer tablet design. This enantioselective advantage provides a strong clinical rationale for replacing racemic amlodipine with S-amlodipine in fixed-dose combinations, as it not only maintains therapeutic efficacy but also addresses safety concerns that limit patient adherence in long-term antihypertensive therapy. [20,21,22,34]. A formulation that minimized the formation of impurities was established through stability testing to ensure the product quality. In addition, in vitro dissolution studies were conducted to demonstrate equivalence with the marketed Sevikar HCT^®^. To establish a robust in vitro–in vivo correlation (IVIVC) for olmesartan medoxomil, an in vitro dissolution study was initially conducted, followed by an in vivo pharmacokinetic study in beagle dogs. These studies evaluated the ability of in vitro data to predict the in vivo performance. Finally, an in vivo pharmacokinetic study in healthy human subjects was performed to assess the bioequivalence of the newly developed formulation and confirm its clinical applicability. This study applied the clinical benefits of S-amlodipine to a widely used FDC anti-hypertensive product to minimize the adverse effects while maintaining therapeutic efficacy. These findings confirm that the new formulation is comparable to the marketed product in terms of both stability and pharmacokinetic performance, supporting its suitability as a viable therapeutic alternative.

## 2. Materials and Methods

### 2.1. Materials

Olmesartan medoxomil (C_29_H_30_N_6_O_6_; molar mass: 558.595 g/mol) was obtained from Pharmacostech (Gyeonggi-do, Republic of Korea). Hydrochlorothiazide (C_7_H_8_ClN_3_O_4_S_2_, molar mass: 297.74 g/mol) was purchased from Polpharma (Sieradz, Poland). S-amlodipine besylate dihydrate (C_26_H_29_ClN_2_O_8_S·2H_2_O, MW: 603.09 g/mol) was sourced from Glochem (Telangana, India). Excipients used in the formulation included colloidal silicon dioxide (Evonik; Essen, Germany), croscarmellose sodium (DFE Pharma GmbH & Co., Goch, Germany), crospovidone (Ashland Inc., Wilmington, DE, USA), low-substituted hydroxypropyl cellulose (Shin-Etsu; Tokyo, Japan), magnesium stearate (FACI Asia Pacific PTE Ltd., Merlimau Pl, Singapore), pregelatinized starch (Colorcon Inc., West Point, PA, USA), silicified microcrystalline cellulose (JRS PHARMA GmbH & Co., Rosenberg, Germany), and Opadry II pink (85F25437; Colorcon Inc., Shanghai, China). A commercial available reference product (Sevikar HCT^®^; 10/40/12.5 mg) was purchased from Daiichi Sankyo Korea, Limited (Seoul, Republic of Korea). High-performance liquid chromatography (HPLC)-grade methanol, ethanol, and acetonitrile were obtained from Honeywell Burdick & Jackson (Muskegon, MI, USA). All experiments were performed using Milli-Q^®^ distilled water. All other chemicals were of reagent grade.

### 2.2. Methods

#### 2.2.1. Compatibility Study Method

We evaluated the compatibility between APIs and excipients, for which each API in Sevikar HCT^®^ tablets was individually mixed in a 1:1 (*w*/*w*) ratio with excipients used in the formulation, as well as with commonly used tablet excipients selected based on their functional roles (e.g., diluents, binders, disintegrants, lubricants, etc.). The binary mixtures were stored under a controlled hot and humid environment at 60 ± 2 °C and 80 ± 5% relative humidity (RH) for up to 4 weeks. The compatibility study was conducted in a temperature and humidity chamber (Sunil Eyela Co., Seongnam, Korea). Samples were withdrawn at predetermined intervals and analyzed for impurities (%).

A HPLC system (Agilent Technologies, Inc. CA, USA) was used for the quantitative analysis of impurities. The column used was Symmetry C8 (100 mm × 4.6 mm, 3.5 μm, Waters Corp., Milford, CT, USA). Mobile phase A consisted of a mixture of phosphate buffer (pH 2.8) and acetonitrile at a ratio of 1700:300 (*v*/*v*) prepared in a 1000 mL volumetric flask. The buffer was prepared by adjusting a 10% phosphoric acid solution to pH 2.8, filtered through a 0.45 μm membrane filter (Millipore; Billerica, MA, USA), and degassed before use. Mobile phase B consisted of a phosphate buffer (pH 2.8) and acetonitrile at a ratio of 600:1400 (*v*/*v*), which was similarly prepared and degassed before use. The mobile phases were delivered at a flow rate of 1.0 mL/min, the column temperature was maintained at 35 °C, and detection was performed at a wavelength of 250 nm.

#### 2.2.2. Preparation of Single-Layer Fixed-Dose Combination Products

Single-layer FDC tablets containing olmesartan medoxomil, hydrochlorothiazide, and amlodipine (S-amlodipine besylate or amlodipine besylate) were prepared using the direct compression method. The components of the formulation are listed in Table 1. The APIs olmesartan medoxomil, hydrochlorothiazide, S-amlodipine besylate, and amlodipine besylate were initially blended with excipients, including pregelatinized starch, silicified microcrystalline cellulose, and croscarmellose sodium, using a bin blender (JSI, Korea) operating at 6 rpm for 5 min to achieve a uniform mixture. After the initial blending, magnesium stearate was added as a lubricant, and the mixture was further blended in the same bin blender at 6 rpm for 3 min to ensure uniform distribution of the lubricant while minimizing the risk of over-lubrication. Tablet compression was performed using a rotary tablet press (PTK, Korea) operated at a turret speed of 30 rpm and a compression force of 1000 kg/cm^2^ under controlled environmental conditions. The core tablets were subsequently coated using a film-coating machine (Geumsung Machinery Co., Ltd., Hwaseong, Republic of Korea) with Opadry^®^ (Colorcon Inc., West Point, PA, USA) in a conventional coating pan until the desired coating weight and uniformity were achieved.

#### 2.2.3. Preparation of Bilayer, Fixed-Dose Combination Products

##### Olmesartan Medoxomil (First Layer), Hydrochlorothiazide and S-Amlodipine Besylate (Second Layer)

Bilayer FDC tablets containing olmesartan medoxomil, hydrochlorothiazide, and S-amlodipine besylate were prepared using a combination of wet and dry granulation methods. The formulation components of each layer are listed in Table 2. In the first layer, olmesartan medoxomil, pre-gelatinized starch, silicified microcrystalline cellulose, and croscarmellose sodium were blended in a bin blender (JSI) at 6 rpm for 5 min. The preblended mixture was subsequently granulated using a high-shear mixer granulator (Geumsung Machinery Co., Ltd., Hwaseong, Republic of Korea) with purified water as the granulating fluid. The wet mass was subsequently dried in a tray dryer, and the dried granules were sized using a cone mill (Hanningfield; UK) to ensure uniform particle distribution. After milling, magnesium stearate was added as a lubricant, and the final blend was gently mixed in the same blender at 6 rpm for 3 min. In the second layer, hydrochlorothiazide, S-amlodipine besylate, pre-gelatinized starch, silicified microcrystalline cellulose, and croscarmellose sodium were blended and processed via dry granulation. The granules were lubricated with magnesium stearate during final blending. The granules prepared for both layers were sequentially compressed into bilayer tablets using a double-sided rotary tablet press (PTK) operated at a turret speed of 25 rpm and a compression force of 1000 kg/cm^2^ under controlled conditions. The resulting bilayer tablets were coated using a film-coating material (Opadry^®^, Colorcon Inc., West Point, PA, USA) in a conventional coating pan (Geumsung Machinery Co., Ltd., Hwaseong, Republic of Korea) until the desired coating weight and uniformity were achieved.

##### Olmesartan Medoxomil and Hydrochlorothiazide (First Layer), S-Amlodipine Besylate (Second Layer)

Bilayer FDC tablets containing olmesartan medoxomil, hydrochlorothiazide, and S-amlodipine besylate were prepared using a combination of wet granulation and direct blending. The formulation components of each layer are listed in Table 3. In the first layer, olmesartan medoxomil, silicified microcrystalline cellulose, pregelatinized starch, and croscarmellose sodium were blended using a bin blender (JSI) at 6 rpm for 5 min. The blended mixture was granulated using a high-shear mixer granulator (Geumsung Machinery Co., Ltd.) with purified water as the granulating fluid. The wet mass was dried in a tray dryer and milled using a cone mill (Hanningfield) to obtain uniform granules. After granulation and sizing, the olmesartan-containing granules were mixed with hydrochlorothiazide, silicified microcrystalline cellulose, and pregelatinized starch in a bin blender. Next, silicon dioxide and magnesium stearate were added, and the mixture was lubricated at 6 rpm for 3 min to complete the preparation of the first layer. In the second layer, S-amlodipine besylate, silicified microcrystalline cellulose, low-substituted hydroxypropyl cellulose (L-HPC), and crospovidone were blended using a bin blender (JSI) at 6 rpm for 5 min. After initial blending, silicon dioxide and magnesium stearate were added, and the mixture was further blended at 6 rpm for 3 min to achieve uniform lubrication. The granules prepared for both layers were sequentially compressed into bilayer tablets using a double-sided rotary tablet press (PTK) operated at a turret speed of 25 rpm and a compression force of 1000 kg/cm^2^ under controlled conditions. The compressed bi-layer tablets were coated using a film-coating material (Opadry^®^) in a conventional coating pan (Geumsung Machinery Co., Ltd.) until the desired coating weight and appearance were achieved.

#### 2.2.4. Product Quality Evaluation

The following tests were performed to assess the quality of the bilayer tablet (B-4): content uniformity, dissolution, and impurity analyses. Content uniformity, assay, and impurity levels of the active ingredients were measured using validated HPLC methods. The in vitro dissolution test was conducted using the USP Apparatus 2 (paddle method) in 900 mL of a phosphate buffer (pH 6.8) at 37.0 ± 0.5 °C with a paddle rotation speed of 50 rpm. At specified time points, samples were withdrawn, filtered through a 0.2 μm membrane filter, and analyzed by HPLC (Table A1).

#### 2.2.5. Stability Method

##### Stability Test Under Stressed Conditions

The stability of single-layer tablets (S-1–S-3), the bilayer tablet (B-4), and Sevikar HCT^®^ was evaluated under stress conditions to assess the formation of impurities over time. The tablets were stored under a controlled hot and humid environment at 60 ± 2 °C and 80 ± 5% RH for up to 4 weeks. The stability tests were conducted in a temperature and humidity chamber (Sunil Eyela Co., Seongnam, Republic of Korea). The samples were withdrawn at predetermined intervals and analyzed for impurities (%) to evaluate the stability of the products under stress conditions. The rate of increase in the total impurity was assessed using the slope *k* obtained from the linear regression analysis to quantitatively evaluate impurity growth under stress conditions. The slope, *k*, representing the rate of total impurity increase (%/week), was calculated using a simple linear regression model based on the following equation:

(1) $$k=\frac{∆I}{∆t}=\frac{I_{t}-I_{0}}{t}$$

where *I_t_* is the total impurity at time *t* (in weeks), *I*_0_ is the initial impurity level, and *t* is the storage duration (in weeks).

An HPLC system (Agilent Technologies Inc., CA, USA) was used for the quantitative analysis of impurities. The column used was Symmetry C8 (100 mm × 4.6 mm, 3.5 μm, Waters Corp., Milford, CT, USA). Mobile phase A consisted of a mixture of phosphate buffer (pH 2.8) and acetonitrile at a ratio of 1700:300 (*v*/*v*) prepared in a 1000 mL volumetric flask. The buffer was prepared by adjusting a 10% phosphoric acid solution to pH 2.8, filtered through a 0.45 μm membrane filter (Millipore), and degassed before use. Mobile phase B consisted of a phosphate buffer (pH 2.8) and acetonitrile at a ratio of 600:1400 (*v*/*v*), which was similarly prepared and degassed before use. The mobile phases were delivered at a flow rate of 1.0 mL/min, the column temperature was maintained at 35 °C, and detection was performed at a wavelength of 250 nm. The gradient elution was programmed as follows: the run started at 100% mobile phase A (0% B) and was maintained isocratically for 5 min. A linear gradient was then applied to reach 0% A (100% B) over 55 min (from 5 to 60 min), followed by an isocratic hold at 100% B for 10 min (60–70 min). The composition was then returned to the initial condition (100% A) within 0.1 min and re-equilibrated at 100% A for 10 min (70.1–80 min) prior to the next injection.

##### Stability Test of Bilayer Tablet Under Accelerated Conditions

The stability of the bilayer tablet (B-4) was evaluated under accelerated conditions in accordance with the ICH guidelines. The tablets were stored at a controlled temperature of 40 ± 2 °C and RH of 75 ± 5% for up to 6 months. Stability tests were performed in a temperature- and humidity-controlled chamber (Sunil Eyela Co., Seongnam, Republic of Korea). The samples were withdrawn at predetermined time points and analyzed for dissolution, assays, and impurities to assess the stability profile of the formulation under hot and humid conditions.

The quantitative analysis method used for both the assay and dissolution tests of olmesartan medoxomil, hydrochlorothiazide, and S-amlodipine besylate was performed using an HPLC system (Agilent Technologies, Inc., Santa Clara, CA, USA). Chromatographic separation was performed on a Capcell Pak C18 column (150 mm × 4.6 mm, 5 μm; Shiseido Co., Ltd., Tokyo, Japan). The mobile phase A consisted of a pH 3.8 phosphate buffer (prepared using 0.015 M phosphoric acid), made up to 1000 mL in a volumetric flask, filtered through a 0.45 μm membrane filter (Millipore), and degassed before use. Mobile phase B consisted of acetonitrile and was prepared in the same manner. The flow rate was set to 1.0 mL/min, the column temperature was maintained at 40 °C, and the detection wavelength was set at 250 nm. The gradient program was as follows: the initial condition was 80% mobile phase A and 20% mobile phase B, maintained isocratically for 3 min. A linear gradient was then applied to reach 55% A and 45% B at 5 min, followed by an isocratic hold at this composition until 11 min. The mobile phase was then returned to 80% A and 20% B within 1 min (at 12 min) and held for re-equilibration until 15 min before the next injection.

#### 2.2.6. In Vitro Test

##### In Vitro Evaluation of Single-Layer Tablet

The in vitro drug release profiles of Sevikar HCT^®^ and the single-layer tablet (S-1) were evaluated in accordance with the USP <711> Dissolution monograph using Apparatus 2 (paddle method). Each tablet was tested in two separate dissolution media, 900 mL of a pH 1.2 buffer and 900 mL of a pH 6.8 buffer, to assess the release characteristics under different physiological conditions. The paddle rotation speed was set to 50 rpm, and the temperature of the dissolution medium was maintained at 37.0 ± 0.5 °C throughout the test. Samples (10 mL) were withdrawn at 5, 10, 15, 30, 45, and 60 min and replaced with an equal volume of fresh, prewarmed buffer to maintain a constant volume. The collected samples were filtered through a 0.2 μm membrane filter before HPLC analysis. Dissolution profile comparison between the test (S-1) and reference (Sevikar HCT^®^, Daiichi Sankyo Co., Ltd., Tokyo, Japan) was conducted in accordance with USP <1090> Assessment of Solid Oral Drug Product Performance and Interchangeability, Bioavailability, Bioequivalence, and Dissolution, specifically the section on Dissolution Profile Comparisons. A similarity factor (*f*_2_) value of 50 or greater (50–100) indicates similarity in dissolution profiles, thereby supporting equivalent in vitro performance between the two formulations [35]. According to the FDA guidelines, at least three dissolution time points were used, with no more than one point exceeding 85% of the labeled amount dissolved. For products that exhibited very rapid dissolution (≥85% within 15 min), profile comparison was deemed unnecessary. The *f*_2_ was calculated using the following equation:

(2) $$f_{2}=50\times log\left\{{\left[1+(\frac{1}{n})\sum_{t=1}^{n}{\left(R_{t}-T_{t}\right)}^{2}\right]}^{-0.5}\times 100\right\}$$

where *R_t_* and *T_t_* are the cumulative percentages of the drug dissolved at each of the selected *n* time points for the *R* and *T* products, respectively.

An HPLC system (Agilent Technologies Inc., Santa Clara, CA, USA) was used for quantitative analysis of olmesartan medoxomil, hydrochlorothiazide, and S-amlodipine besylate. The column used was Capcell Pak C18 (150 mm × 4.6 mm, 5 μm, Shiseido Co., Ltd., Tokyo, Japan). The mobile phase A consisted of a pH 3.8 phosphate buffer (adjusted with a 0.015 M phosphoric acid) in a 1000 mL volumetric flask, filtered through a 0.45 μm membrane filter (Millipore), and further degassed before use. Mobile phase B consisted of acetonitrile in a 1000 mL volumetric flask and was further degassed before use. The mobile phase was pumped through the column at a 1.0 mL/min flow rate. The column temperature was set to 40 °C, and the detection wavelength was 250 nm. The mixing conditions for mobile phases A and B were the same as those described in the section entitled Stability Test of Bilayer Tablet Under Accelerated Conditions.

##### In Vitro Evaluation of Bi-Layer Tablet

The in vitro drug release profiles of Sevikar HCT^®^ and the bi-layer tablets (B-1–B-4) were evaluated in accordance with the USP <711> Dissolution monograph using Apparatus 2 (paddle method). Each tablet was tested in two separate dissolution media: 900 mL of a pH 1.2 buffer and 900 mL of a pH 6.8 buffer. The paddle rotation speed was maintained at 50 rpm, and the dissolution medium was kept at a constant temperature of 37.0 ± 0.5 °C. At 5, 10, 15, 30, 45, and 60 min, 10 mL samples were withdrawn and immediately replaced with an equal volume of fresh pre-warmed buffer to maintain a constant volume. The withdrawn samples were filtered through a 0.2 μm membrane filter before HPLC.

An HPLC system (Agilent Technologies Inc., Santa Clara, CA, USA) was used for quantitative analysis of olmesartan medoxomil, hydrochlorothiazide, and S-amlodipine besylate. The column used was Capcell Pak C18 (150 mm × 4.6 mm, 5 μm, Shiseido Co., Ltd., Japan). The mobile phase A consisted of a pH 3.8 phosphate buffer (adjusted with a 0.015M phosphoric acid) in a 1000 mL volumetric flask, filtered through a 0.45 μm membrane filter (Millipore), and further degassed before use. Mobile phase B consisted of acetonitrile in a 1000 mL volumetric flask and was further degassed before use. The mobile phase was pumped through the column at a 1.0 mL/min flow rate. The column temperature was set to 40 °C, and the detection wavelength was 250 nm. The mixing conditions for mobile phases A and B were the same as those described in the section entitled Stability Test of Bilayer Tablet Under Accelerated Conditions.

#### 2.2.7. In Vivo Pharmacokinetic Study

##### In Vivo Pharmacokinetic Study of Single-Layer Tablet

A randomized, open-label, two-group, two-period, single-dose, crossover study was conducted to evaluate and compare the pharmacokinetic (PK) characteristics of the test (single-layer tablet, S-1) and the reference (Sevikar HCT^®^) following oral administration in 12 healthy male beagle dogs (19–20 months old, Marshall Biotechnology Co., Ltd., Beijing, China). Each dog received a single dose of the test or reference formulation, administered by placing one tablet at the root of the tongue, followed by forced delivery into the stomach using 10–20 mL of water. A washout period of 1 week was observed between the two dosing periods. The dogs were fasted from 17:00 to 18:00 on the day before drug administration, and food was provided approximately 4 h after dosing. Blood samples were collected from each dog at 16 time points: pre-dose (0 h) and at 0.25, 0.5, 1, 1.5, 2, 2.5, 3, 3.5, 4, 5, 6, 8, 12, 16, and 24 h post-dose to determine the plasma concentrations of S-amlodipine besylate, olmesartan medoxomil, and hydrochlorothiazide. Pharmacokinetic parameters, including the area under the concentration-time curve (AUC_0-t_) and the maximum plasma concentration (C_max_), were calculated using non-compartmental analysis. Statistical comparisons of pharmacokinetic parameters were conducted using a generalized linear mixed-effects model (GLMM) with log-transformed values of AUC_0-t_ and C_max_. The model included sequence, period, and treatment as fixed effects, and subject nested within the sequence as a random effect. The significance level was set at *p* < 0.05. To compare the formulations, the least squares mean difference and 90% confidence interval (CI) were calculated, and the geometric mean ratio (GMR) and its 90% CI were obtained by exponentiating the log-transformed data. Bioequivalence was considered if the 90% CI for the GMR of both the AUC_0-t_ and C_max_ was within the accepted range of 0.80–1.25. This study was conducted at Dt&CRO (Gyeonggi-do, Republic of Korea), in compliance with the Animal Protection Act and related regulations. The study protocol was reviewed and approved by the Institutional Animal Care and Use Committee (IACUC) of the testing facility (approval no. 200068).

##### In Vivo Pharmacokinetic Study of Bi-Layer Tablet

A randomized, open-label, two-group, two-period, single-dose, crossover study was conducted to compare and evaluate the PK characteristics of the test (bi-layer tablet, B-4) and the reference (Sevikar HCT^®^) in 38 healthy adult volunteers aged between 19 and 45 years, with a body mass index in the range of 18.5 to 27.0 kg/m^2^ and a body weight of at least 50 kg. Each subject received a single oral dose of either the test or reference product, followed by a washout period of 1 week, and subsequently received the alternate formulation. Blood samples were collected to determine the PK profiles of S-amlodipine besylate, olmesartan medoxomil, and hydrochlorothiazide. For the pharmacokinetic analysis, blood samples were collected at different time points appropriate for each drug component. For S-amlodipine besylate, blood was drawn just before dosing (0 h) and at 1, 2, 3, 4, 5, 6, 7, 8, 10, 12, 24, 48, and 72 h after administration. For olmesartan medoxomil, samples were collected at 0 (pre-dosing) and 0.5, 1, 1.5, 2, 2.5, 3, 4, 6, 8, 12, 24, and 48 h post-dosing. For hydrochlorothiazide, sampling was conducted at 0 h (pre-dose) and subsequently at 0.5, 1, 1.5, 2, 2.5, 3, 4, 6, 8, 10, 12, 24, and 48 h after administration. Pharmacokinetic analysis was performed to calculate the AUC_0-t_ and C_max_ for each drug component. Descriptive statistics, including means and standard deviations, were calculated for each treatment group. For statistical analysis, AUC_0-t_ and C_max_ values were log-transformed, and comparisons between the test and reference products were made using a GLMM analysis of variance. The model included sequence, period, and treatment as fixed effects, and subject nested within the sequence as a random effect. Statistical significance was set at α = 0.05. Differences in the least-squares means between the formulations and associated 90% CIs were obtained. These exponentiations were used to derive the GMR and 90% CI. Bioequivalence was determined if the 90% CI of the GMR for both the AUC_0-t_ and C_max_ fell within an equivalence margin of 0.80–1.25. This study was conducted at Gil Medical Center (Incheon, Korea) in compliance with the principles of the Declaration of Helsinki and Good Clinical Practice. The clinical protocol was reviewed and approved by the Institutional Review Board, and written informed consent was obtained from all participants prior to enrollment.

## 3. Results

### 3.1. Compatibility Study

Compatibility studies were conducted between each API and the selected excipients to minimize the formation of degradation products during formulation development. For S-amlodipine besylate, a notable increase in total impurities was observed when combined with certain excipients under stressed conditions (60 ± 2 °C, 80 ± 5% RH) over a period of 4 weeks. Specifically, the total impurity levels increased by 1.53% with mannitol, 0.81% with croscarmellose sodium, and 0.65% with both sodium starch glycolate and povidone (Table A2). In contrast, olmesartan medoxomil and hydrochlorothiazide demonstrated no significant increase in the related impurities when combined with the tested excipients under the same conditions (Table A3 and Table A4). Based on these results, excipients that increased impurity levels when combined with S-amlodipine besylate were excluded from further formulation development to enhance the stability of the final dosage form.

### 3.2. Stability

#### 3.2.1. Stability Testing Under Stress Conditions

**Figure 1:**
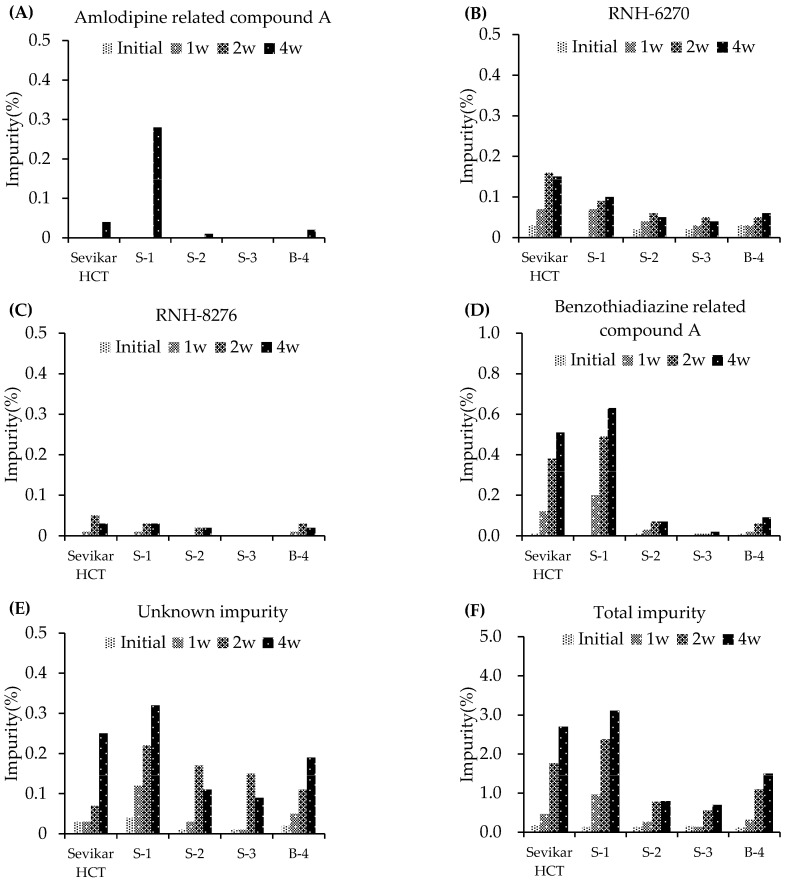
Stability testing of single-layer and bilayer fixed-dose combination products under stress conditions. (**A**) Amlodipine-related compound A. (**B**) Olmesartan (RNH-6270). (**C**) Olmesartan dimer ester (RNH-8276). (**D**) Benzothiadiazine-related compound A. (**E**) Unknown impurity. (**F**) Total impurity.

A stability study was conducted to evaluate the impurity profiles of three single-layer tablet formulations (S-1–S-3), the bilayer table (B-4), and the marketed product Sevikar HCT^®^ under stressed conditions (60 ± 2 °C, 80 ± 5% RH) for 4 weeks (Figure 1). In Sevikar HCT^®^, the olmesartan-related impurity RNH-6270 increased to 0.15%, benzothiadiazine-related compound A to 0.51%, and unknown impurities to 0.25%, with total impurities reaching 2.70% by week 4. Formulation S-1, which contained olmesartan medoxomil, hydrochlorothiazide, and S-amlodipine besylate, exhibited the highest degradation among all tested formulations. Specifically, amlodipine-related compound A increased to 0.28%, benzothiadiazine-related compound A increased to 0.63%, unknown impurities increased to 0.32%, and total impurities increased to 3.11% over 4 weeks. Formulation S-2, in which racemic amlodipine besylate was used instead of S-amlodipine besylate, showed improved stability. The level of unknown impurities increased from 0.01% to 0.11% over 4 weeks, which was approximately 0.21% lower than that observed in S-1 and remained within acceptable limits. Formulation S-3, excluding amlodipine besylate, exhibited an impurity trend similar to that of S-2, with unknown impurities increasing from 0.01% to 0.09% by week 4. Formulation B-4 is a bilayer tablet in which olmesartan medoxomil and hydrochlorothiazide are compressed in the first layer, whereas S-amlodipine besylate is separately compressed in the second layer. In contrast to the single-layer formulation S-1, in which all three APIs were coformulated, B-4 exhibited improved stability profiles under stress conditions. Amlodipine-related compound A, a known impurity of S-amlodipine besylate, increased by only 0.02% over 4 weeks, indicating significant suppression of impurity formation when S-amlodipine besylate was isolated from the second layer. Similarly, the known impurities in olmesartan medoxomil remained virtually unchanged and were comparable to the levels observed in other formulations. The known hydrochlorothiazide impurity, benzothiadiazine-related compound A, increased by 0.09% over 4 weeks, which was markedly lower than the increase observed in the S-1 formulation, indicating superior stability. The levels of unknown impurities were comparable to those of the other formulations. Overall, the total impurities increased by approximately 1.5% over 4 weeks, which was significantly lower than that of the single-layer formulation of S-1. These results suggest that the bilayer design of formulation B-4 effectively improved the stability of the product by minimizing interaction-induced degradation between the APIs.

To quantitatively evaluate the rate of total impurity formation under stress conditions, the slope *k* (%/week) was calculated for each formulation based on the total impurity levels measured over 4 weeks (Table 4). Linear regression was applied to the impurity measurements collected at 0, 1, 2, and 4 weeks to estimate the best-fit slope, which represents the average weekly increase in the impurity content under stress conditions. Among the formulations, S-1 exhibited the highest degradation rate with a calculated slope of 0.757%/week, indicating the lowest stability. In contrast, bilayer formulation B-4, in which S-amlodipine besylate was physically separated from olmesartan medoxomil and hydrochlorothiazide, demonstrated a significantly lower slope of 0.366%/week. This suggests that the separation of APIs in B-4 effectively reduces impurity generation, thereby enhancing formulation stability.

These findings indicate that the combination of S-amlodipine besylate with olmesartan medoxomil and hydrochlorothiazide in a single-layer tablet formulation may compromise the stability. Therefore, a bilayer tablet design that separates S-amlodipine besylate from other APIs is recommended to enhance formulation stability.

#### 3.2.2. Stability Testing Under Accelerated Conditions

**Figure 2:**
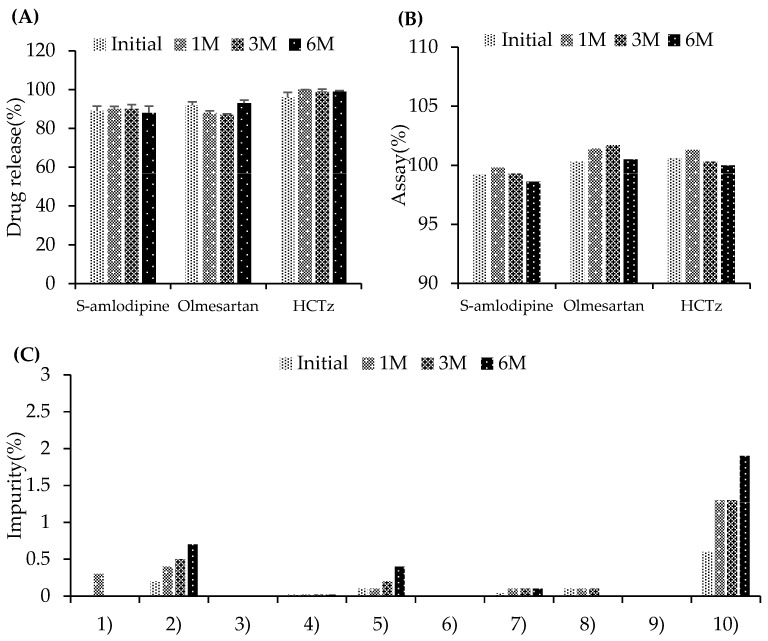
Stability testing of a bilayer, fixed-dose combination product under accelerated conditions. (**A**) Dissolution. (**B**) Assay. (**C**) Impurity (1) amlodipine related compound A, (2) olmesartan (RNH-6270), (3) olmesartan dimer ester (RNH-8276), (4) olefinic impurity (RNH-6373), (5) benzothiadiazine-related compound A, (6) unknown impurity derived from S-amlodipine, (7) unknown impurity derived from olmesartan medoxomil, (8) unknown impurity derived from hydrochlorothiazide, (9) unknown impurity, and (10) total impurity.

The B-4 formulation, selected based on in vitro dissolution performance, was subjected to accelerated stability testing at 40 ± 2 °C and 75 ± 5% RH for 6 months. In the dissolution test, S-amlodipine besylate showed a slight change in release, with a dissolution rate of 89% at the initial time point and 88% after six months under accelerated conditions, indicating consistent performance. Olmesartan medoxomil exhibited a dissolution rate of 92% initially and 93% after 6 months, whereas that of hydrochlorothiazide increased from 96% to 99%, maintaining stable and acceptable dissolution profiles (Figure 2A). The S-amlodipine besylate content decreased slightly from 99.2% to 98.6% after 6 months. Olmesartan medoxomil slightly increased from 100.3% to 100.5%, and hydrochlorothiazide slightly decreased from 100.6% to 100.0%. All three APIs remained within acceptable specification limits throughout the 6-month stability study (Figure 2B). In the impurity profile, the known impurity of olmesartan (RNH-6270) increased from 0.2% at the initial time point to 0.7% at 6 months, which remained within the acceptance criterion of 2.5%. For hydrochlorothiazide, the known impurity (benzothiadiazine-related compound A) increased from 0.1% to 0.4%, which was also within the acceptance criterion of 0.7%. Other impurities exhibited only minor increases. The total impurities increased from 0.6% to 1.35% at 6 months, which remained well below the acceptance criterion of 5.0% (Figure 2C). Overall, the results of the dissolution, assay, and impurity tests confirmed that the B-4 formulation remained stable and met the specified criteria under accelerated conditions over 6 months.

### 3.3. In Vitro Test

#### 3.3.1. Dissolution Test of Single-Layer Tablet

**Figure 3:**
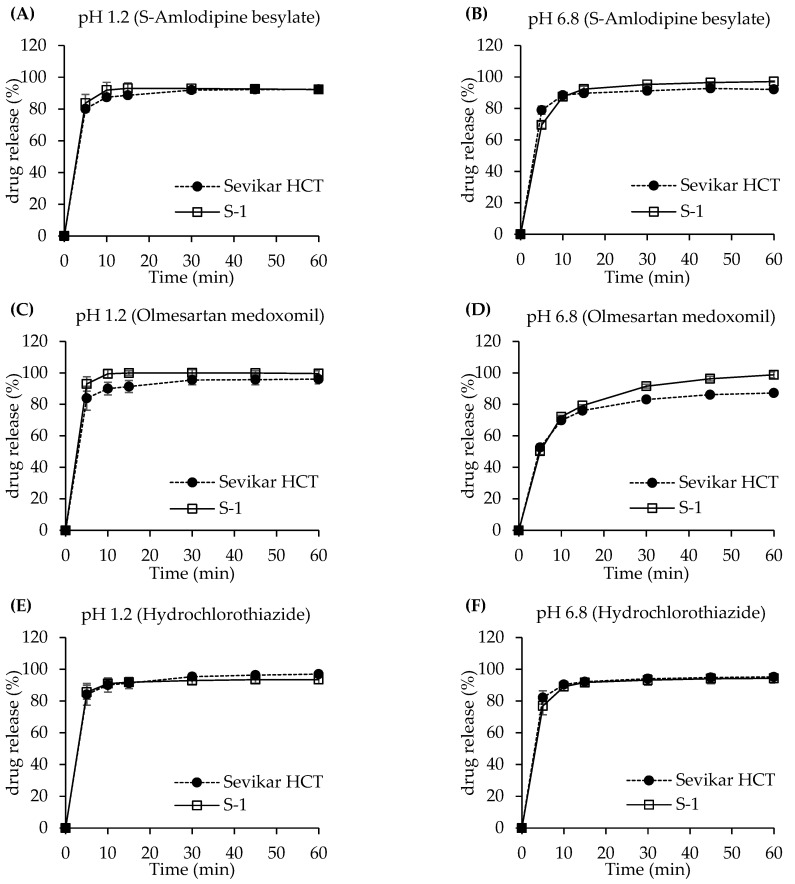
Dissolution profiles of the single-layer tablet (S-1) and the reference tablet. (**A**) S-amlodipine besylate in pH 1.2. (**B**) S-amlodipine besylate in pH 6.8. (**C**) Olmesartan medoxomil in pH 1.2. (**D**) Olmesartan medoxomil in pH 6.8. (**E**) Hydrochlorothiazide in pH 1.2. (**F**) Hydrochlorothiazide in pH 6.8.

The dissolution profiles of the single-layer tablet formulation S-1 were compared with those of the reference (Sevikar HCT^®^) in media of pH 1.2 and pH 6.8, as shown in Figure 3. For S-amlodipine besylate, the dissolution at pH 1.2 demonstrated that the test (S-1) exhibited approximately 3% higher dissolution than the reference tablet at 5 min, 4% higher at 10 min, and 5% higher at 15 min. Beyond 15 min, the dissolution rates of the test tablet and reference (Sevikar HCT^®^) formulations were comparable. At pH 6.8, the reference (Sevikar HCT^®^) displayed about 9% higher dissolution at 5 min, 1% higher at 10 min, and 3–5% higher at 15 min compared to the test (S-1), after which the dissolution profiles became similar. For olmesartan medoxomil, at pH 1.2, the test (S-1) exhibited approximately 10% higher dissolution than the reference (Sevikar HCT^®^) at both 5 and 10 min. After 15 min, the difference in the dissolution rates gradually decreased, resulting in only a 3% difference at 60 min. At pH 6.8, a 2–3% higher dissolution rate was observed for test (S-1) during the initial 10 min. However, the difference increased with time, and at 60 min, the test (S-1) demonstrated approximately 10% higher dissolution rate than the reference (Sevikar HCT^®^). For hydrochlorothiazide, dissolution rate results at pH 1.2 indicated minimal difference (about 1%) between the two tablets from 5 to 15 min, whereas the reference (Sevikar HCT^®^) showed approximately 4% higher dissolution rate at 60 min. At pH 6.8, the reference (Sevikar HCT^®^) demonstrated about 5% higher dissolution rate at 5 min; however, the difference between the two tablets decreased over time, resulting in only about a 1% difference after 10 min. Overall, although a dissolution rate difference of approximately 10% was observed for olmesartan medoxomil at 60 min in pH 6.8 medium, the test (S-1) demonstrated similar dissolution rate behavior to the reference (Sevikar HCT^®^) across all components and conditions.

Based on USP <1090> and the FDA guidance, dissolution testing was conducted in media of pH 1.2 and pH 6.8 to assess the in vitro performance of the test (S-1) and reference (Sevikar HCT^®^). Both products demonstrated rapid dissolution, with more than 85% of the drug released within 15 min in pH 1.2 and pH 6.8 media, indicating similarity and equivalence without the need for further profile comparisons. However, for olmesartan medoxomil (pH 6.8 medium), drug release did not reach 85% within 15 min. Consequently, the *f*_2_ was calculated and found to be 51.9, confirming that the test and reference products exhibited similar dissolution profiles in this medium.

#### 3.3.2. Dissolution Test of Bi-Layer Tablet

**Figure 4:**
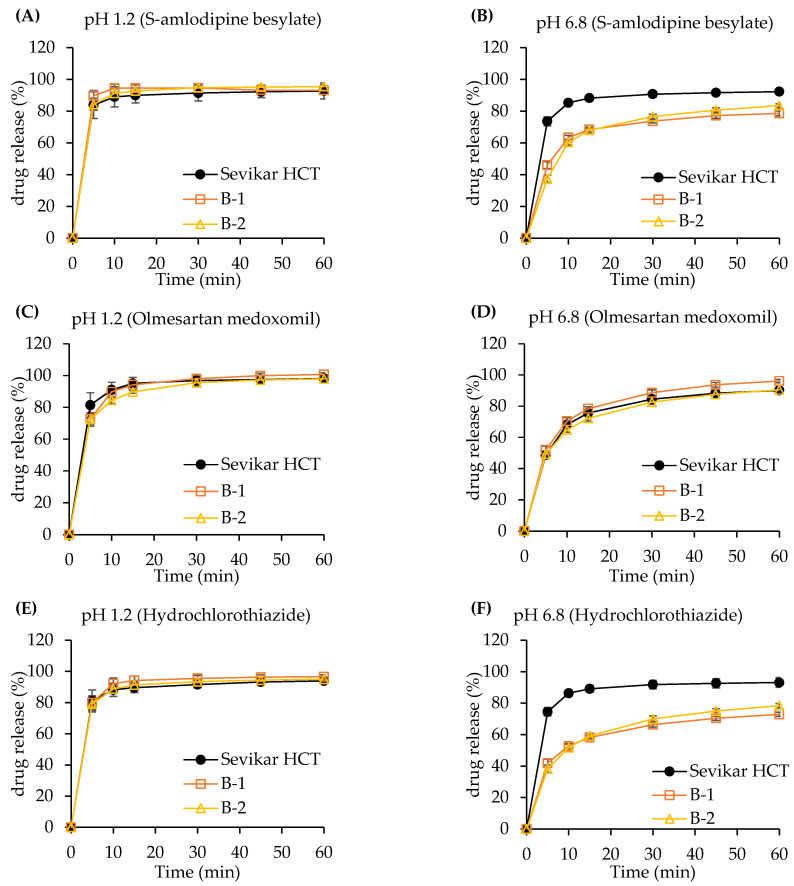
Dissolution profiles of bilayer tablets (B-1, B-2) and the reference tablet. (**A**) S-amlodipine besylate in pH 1.2. (**B**) S-amlodipine besylate in pH 6.8. (**C**) Olmesartan medoxomil in pH 1.2. (**D**) Olmesartan medoxomil in pH 6.8. (**E**) Hydrochlorothiazide in pH 1.2. (**F**) Hydrochlorothiazide in pH 6.8.

**Figure 5:**
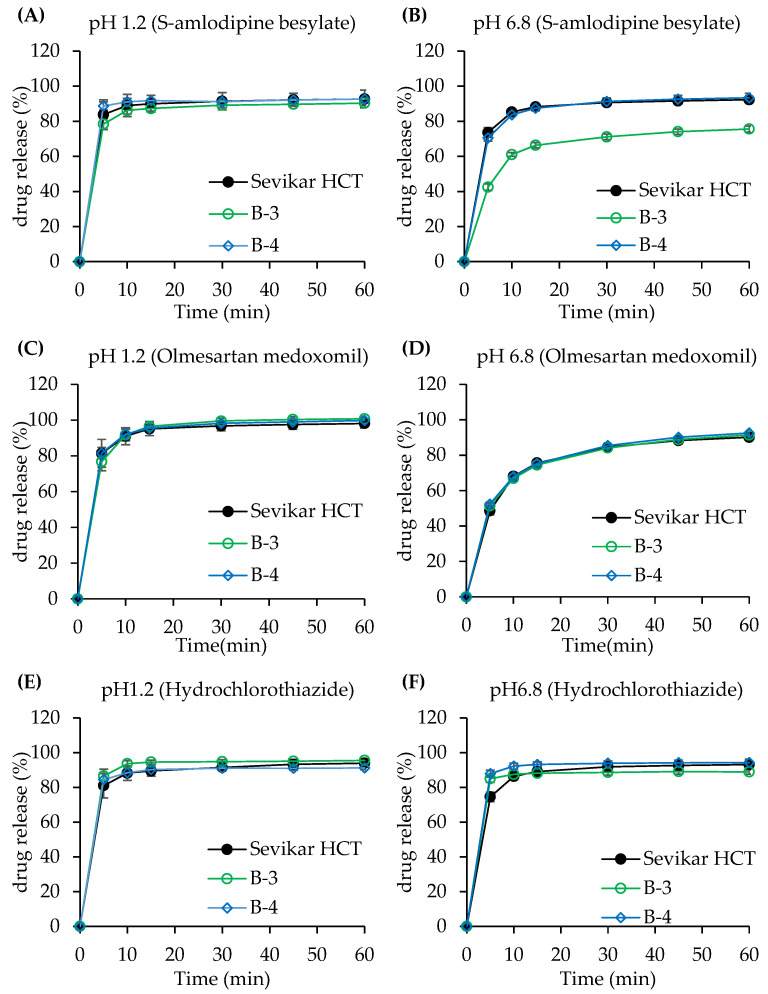
Dissolution profiles of bilayer tablets (B-3, B-4) and the reference tablet. (**A**) S-amlodipine besylate in pH 1.2. (**B**) S-amlodipine besylate in pH 6.8. (**C**) Olmesartan medoxomil in pH 1.2. (**D**) Olmesartan medoxomil in pH 6.8. (**E**) Hydrochlorothiazide in pH 1.2. (**F**) Hydrochlorothiazide in pH 6.8.

The bilayer tablet formulations B-1 and B-2, as presented in Table 2, were manufactured with olmesartan medoxomil in the first layer, and hydrochlorothiazide and S-amlodipine besylate in the second layer. The dissolution profiles of the formulations are shown in Figure 4. Formulations B-3 and B-4, described in Table 3, were composed of olmesartan medoxomil and hydrochlorothiazide in the first layer, and S-amlodipine besylate in the second layer. The dissolution profiles are shown in Figure 5. For formulation B-1, the dissolution rate of S-amlodipine besylate in the pH 1.2 medium showed higher dissolution rates from the test (B-1) compared to that of the reference (Sevikar HCT^®^), with approximately 6%, 5%, 4%, 3%, and 1% at 5, 10, 15, 30, 45, and 60 min, respectively. At pH 6.8, the reference (Sevikar HCT^®^) exhibited higher dissolution rates with about 27%, 23%, 20%, 17%, and 14% at 5, 10, 15, 30, 45, and 60 min, respectively. For olmesartan medoxomil in pH 1.2, the reference (Sevikar HCT^®^) showed about 9% higher dissolution rate at 5 min, whereas the dissolution rate difference was approximately 2% after 10 min. At pH 6.8, test (B-1) exhibited approximately 4–5% higher dissolution rates across all time points. Hydrochlorothiazide in pH 1.2 showed 1% higher dissolution rate in the reference (Sevikar HCT^®^) at 5 min, whereas the test (B-1) had 3–5% higher dissolution rate from 10 min onward. At pH 6.8, the reference (Sevikar HCT^®^) showed greater dissolution rates, with approximately 33%, 33%, 30%, 25%, 22%, and 20% at 5, 10, 15, 30, 45, and 60 min, respectively. In formulation B-2, the test (B-2) of S-amlodipine besylate demonstrated slightly higher dissolution rates (1–3%) at pH 1.2 throughout 5–60 min. However, at pH 6.8, the reference (Sevikar HCT^®^) showed significantly higher dissolution rates with approximately 36%, 25%, 20%, 14%, and 10% at 5, 10, 15, 30, 45, and 60 min, respectively. For olmesartan medoxomil in pH 1.2, the reference (Sevikar HCT^®^) showed 9%, 6%, and 6% higher dissolution rates at 5, 10, and 15 min, respectively, with about 1% higher dissolution rate from 30 min onward. At pH 6.8, test (B-2) and reference (Sevikar HCT^®^) showed similar dissolution rates with a difference of about 1–3%. In addition, hydrochlorothiazide in pH 1.2 showed minimal differences (1–2%) between tablets, whereas in pH 6.8, the reference (Sevikar HCT^®^) showed higher dissolution rates (approximately 36%, 30%, 22%, 17%, and 14% at 5, 15, 30, 45, and 60 min, respectively). In formulation B-3, the reference (Sevikar HCT^®^) of S-amlodipine besylate showed approximately 3% higher dissolution rates than the test (B-3) in pH 1.2. In pH 6.8, the reference (Sevikar HCT^®^) showed higher dissolution rates with 31%, 24%, 22%, 20%, and 17% at 5, 10, 15, 30, 45, and 60 min, respectively. Olmesartan medoxomil dissolution rates in both pH 1.2 and 6.8 conditions showed a difference of 1–4% between the test (B-3) and reference (Sevikar HCT^®^). Hydrochlorothiazide at pH 1.2 exhibited an approximately 5% difference up to 15 min, with a smaller difference rate (1–3%) from 30 min onward. At pH 6.8, the test (B-3) exhibited about 10% higher dissolution rate at 5 min, and a 1–4% difference compared to the reference (Sevikar HCT^®^) thereafter. In formulation B-4, S-amlodipine besylate in pH 1.2 showed 1–4% higher dissolution rate in the test (B-4) throughout the time points, whereas at pH 6.8, test (B-4) and reference (Sevikar HCT^®^) showed similar dissolution rates with differences of approximately 1–3%. Olmesartan medoxomil dissolution rates was also similar between test (B-4) and reference (Sevikar HCT^®^) in both media (1–3% difference). Hydrochlorothiazide at pH 1.2 showed comparable dissolution rates (1–3% difference), whereas at pH 6.8, test (B-4) showed a 13% higher dissolution rate at 5 min, followed by a 1–5% difference over 60 min.

Based on USP <1090> and the FDA guidance, dissolution testing was conducted in media of pH 1.2 and pH 6.8 to assess the in vitro performance of the test (B-4) and reference (Sevikar HCT^®^). Both products demonstrated rapid dissolution, with more than 85% of the drug released within 15 min in pH 1.2 and pH 6.8 media, indicating similarity and equivalence without the need for further profile comparisons. However, for olmesartan medoxomil (pH 6.8 medium), the drug release did not reach 85% within 15 min. Consequently, the *f*_2_ was calculated and found to be 92.5, confirming that the test (B-4) and reference (Sevikar HCT^®^) exhibited similar dissolution profiles in this medium.

### 3.4. Pharmacokinetics

#### 3.4.1. In Vivo Beagle Pharmacokinetic Study

**Figure 6:**
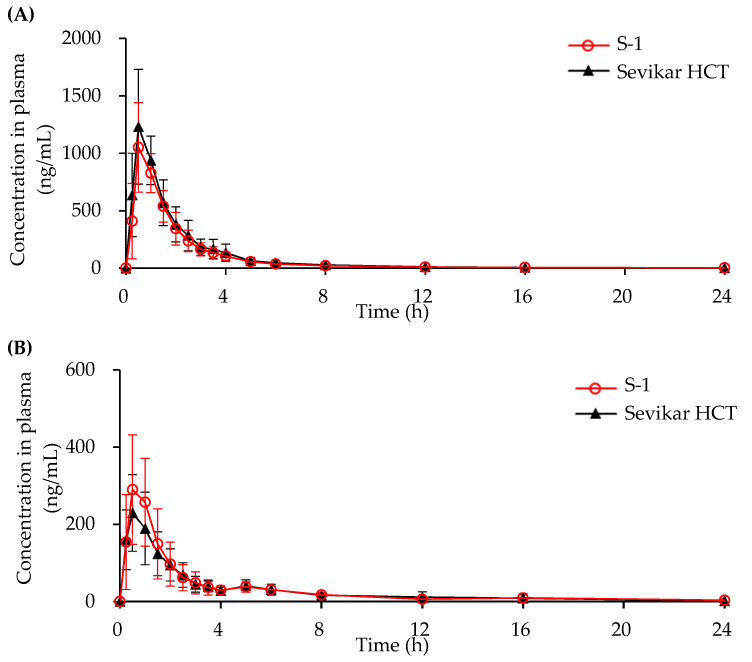

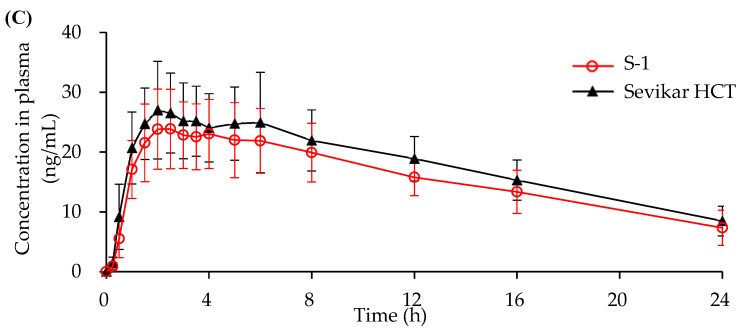
Mean plasma concentration-time profiles in beagle dogs after a single oral administration of (red line) and Sevikar HCT^®^ (black line). (**A**) Hydrochlorothiazide. (**B**) Olmesartan medoxomil. (**C**) S-amlodipine besylate.

The pharmacokinetics following the oral administration of the test tablet (S-1) and the commercial product (Sevikar HCT^®^) at equivalent doses in beagle dogs are presented in Figure 6, and the corresponding pharmacokinetic parameters are summarized in Table 5. The AUC_0-t_ values of hydrochlorothiazide for S-1 and Sevikar HCT were 1902.0 ± 378.8 ng/mL and 2227.2 ± 617.7 ng/mL, respectively. The AUC_0-t_ values of olmesartan medoxomil for S-1 and Sevikar HCT were 695.3 ± 216.5 ng/mL and 639.6 ± 210.1 ng/mL, respectively. The AUC_0-t_ values of s-amlodipine besylate for S-1 and Sevikar HCT were 369.1 ± 85.7 ng/mL and 422.3 ± 87.6 ng/mL, respectively. The *C*_max_ values of hydrochlorothiazide for S-1 and Sevikar HCT were 1131.5 ± 318.5 ng/mL and 1320.3 ± 363.0 ng/mL, respectively. The *C*_max_ values of olmesartan medoxomil for S-1 and Sevikar HCT were 367.5 ± 108.3 ng/mL and 266.3 ± 78.7 ng/mL, respectively. The *C*_max_ values of s-amlodipine besylate for S-1 and Sevikar HCT were 26.5 ± 6.5 ng/mL and 29.7 ± 8.1 ng/mL, respectively. The *T*_max_ values of hydrochlorothiazide for S-1 and Sevikar HCT were 0.67 ± 0.24 h and 0.67 ± 0.24 h, respectively. The *T*_max_ values of olmesartan medoxomil for S-1 and Sevikar HCT were 0.67 ± 0.29 h and 0.55 ± 0.30 h, respectively. The *T*_max_ values of s-amlodipine besylate for S-1 and Sevikar HCT were 3.60 ± 1.72 h and 3.0 ± 1.2 h, respectively. The *T*_1/2_ values of hydrochlorothiazide for S-1 and Sevikar HCT were 4.83 ± 2.81 h and 4.17 ± 1.27 h, respectively. The *T*_1/2_ values of olmesartan medoxomil for S-1and Sevikar HCT were 4.21 ± 1.90 h and 4.12 ± 4.11 h, respectively. The T_1/2_ values of S-amlodipine besylate for S-1 and Sevikar HCT were 10.91 ± 3.07 h and 10.71 ± 2.63 h, respectively.

The geometric mean ratios (tests/references) with 90% Cis from the bioequivalence study are summarized in Table 6. For hydrochlorothiazide, the AUC_0-t_ T/R ratio was 0.85, with a 90% CI of 0.81–0.92, and the C_max_ T/R ratio was 0.86, with a 90% CI of 0.75–0.99. For olmesartan medoxomil, the AUC_0-t_ T/R ratio was 1.08 (90% CI: 0.96–1.21), and the C_max_ T/R ratio was 1.38 (90% CI: 1.13–1.64). For S-amlodipine besylate, the AUC_0-t_ T/R ratio was 0.87 (90% CI: 0.84–0.90), and the C_max_ T/R ratio was 0.89 (90% CI: 0.85–0.95).

#### 3.4.2. In Vivo Human Pharmacokinetic Study

**Figure 7:**
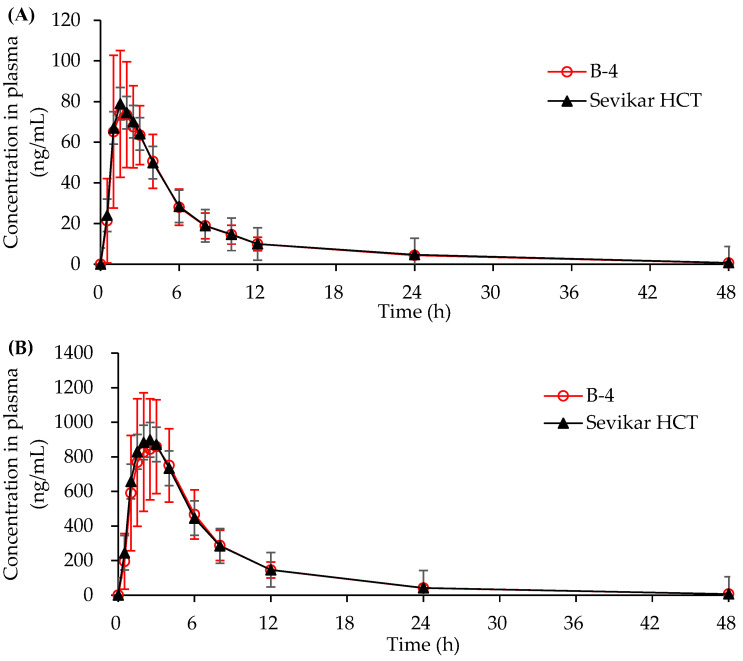

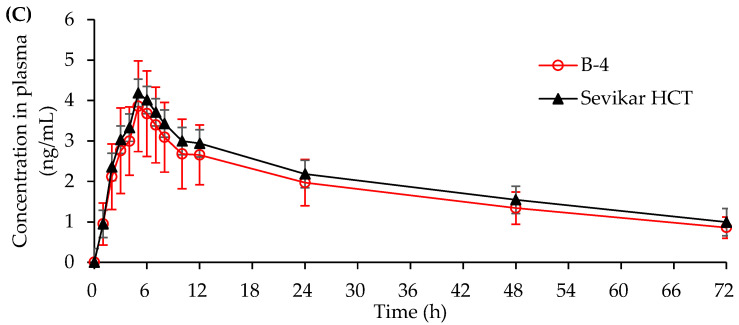
Mean plasma concentration–time profiles in humans after a single oral administration of B-4 (red line) and Sevikar HCT^®^ (black line). (**A**) Hydrochlorothiazide. (**B**) Olmesartan medoxomil. (**C**) S-amlodipine besylate.

The pharmacokinetics following oral administration of the test tablet (B-4) and the commercial product (Sevikar HCT^®^) at equivalent doses in healthy humans are presented in Figure 7. The corresponding pharmacokinetic parameters are summarized in Table 7. The AUC_0-t_ values of hydrochlorothiazide for B-4 and Sevikar HCT were 524.29 ± 150.48 ng/mL and 525.16 ± 129.75 ng/mL, respectively. The AUC_0-t_ values of olmesartan medoxomil for B-4 and Sevikar HCT were 6825.01 ± 1929.15 ng/mL and 6913.78 ± 2105.56 ng/mL, respectively. The AUC_0-t_ values of S-amlodipine besylate for B-4 and Sevikar HCT were 125.49 ± 34.10 ng/mL and 140.81 ± 41.32 ng/mL, respectively. The *C*_max_ values of hydrochlorothiazide for B-4 and Sevikar HCT were 87.59 ± 26.69 ng/mL and 89.42 ± 23.74 ng/mL, respectively. The *C*_max_ values of olmesartan medoxomil for B-4 and Sevikar HCT were 963.13 ± 294.25 ng/mL and 987.24 ± 300.80 ng/mL, respectively. The *C*_max_ values of S-amlodipine besylate for B-4 and Sevikar HCT were 3.99 ± 1.08 ng/mL and 4.32 ± 1.20 ng/mL, respectively. *T*_max_ values of hydrochlorothiazide for B-4 and Sevikar HCT were 1.5 h and 1.5 h, respectively. The *T*_max_ values of olmesartan medoxomil for B-4 and Sevikar HCT were 2.5 h and 2.0 h, respectively. The *T*_max_ values of S-amlodipine besylate for B-4 and Sevikar HCT were 5.0 h and 5.0 h, respectively. The *T*_1/2_ values of hydrochlorothiazide for B-4 and Sevikar HCT were 9.30 ± 1.50 h and 9.42 ± 1.30 h, respectively. The *T*_1/2_ values of olmesartan medoxomil for B-4 and Sevikar HCT were 7.94 ± 1.72 h and 8.01 ± 1.74 h, respectively. The *T*_1/2_ values of S-amlodipine besylate for B-4 and Sevikar HCT were 39.92 ± 8.58 h and 40.31 ± 7.20 h, respectively.

The geometric mean ratios (test/reference) with 90% CIs from the bioequivalence study are summarized in Table 8. For hydrochlorothiazide, the AUC_0-t_ T/R ratio was 0.99, with a 90% CI of 0.95–1.04, and the C_max_ T/R ratio was 0.97, with a 90% CI of 0.90–1.05. For olmesartan medoxomil, the AUC_0-t_ T/R ratio was 1.00 (90% CI: 0.94–1.05), and the C_max_ T/R ratio was 0.98 (90% CI: 0.92–1.05). For S-amlodipine besylate, the AUC_0-t_ T/R ratio was 0.90 (90% CI: 0.86–0.93), and the C_max_ T/R ratio was 0.92 (90% CI: 0.89–0.96).

## 4. Discussion

We developed a stable FDC antihypertensive formulation by replacing amlodipine besylate with its active enantiomer, S-amlodipine besylate, in Sevikar HCT^®^, and evaluated its equivalence in efficacy. The initial formulations (S-1, S-2, and S-3) were prepared as single-layer tablets containing a combination of olmesartan medoxomil, hydrochlorothiazide, and either amlodipine besylate or S-amlodipine besylate. Stability assessments, including stress conditions, revealed that the S-1 formulation, which incorporated S-amlodipine besylate, exhibited a significant increase in unknown impurities, indicating reduced stability compared with the S-2 and S-3 formulations lacking S-amlodipine besylate. These findings suggest that the incorporation of S-amlodipine besylate into a single-layer tablet may not be suitable from the perspective of stability. To address the stability issues associated with S-amlodipine besylate, a bilayer tablet approach was adopted to separate it from other APIs. Different bilayer formulations (B-1 to B-4) were developed that differed in the distribution of API between the two layers. Dissolution testing demonstrated that the B-4 formulation achieved dissolution profiles comparable to Sevikar HCT^®^ for all APIs. Furthermore, the B-4 formulation maintained acceptable stability under accelerated conditions over six months, meeting the criteria for dissolution, assay, and impurity. Specifically, in the stability study under stress conditions, the impurity growth rate, as represented by slope k, was substantially lower for B-4 than for the single-layer formulation S-1. This finding suggests that the physical separation of S-amlodipine into a second layer can effectively minimize the degradation pathways that may arise when all APIs are co-formulated in a single-layer tablet.

In the present bioequivalence study conducted in beagle dogs, the pharmacokinetic parameters of all APIs were evaluated based on the GMRs and their corresponding 90% CIs of the test (S-1) and reference (Sevikar HCT^®^). For hydrochlorothiazide, both the AUC_0-t_ and C_max_ GMRs (0.85 and 0.86, respectively) were within or near the lower bound of the accepted bioequivalence range (0.80–1.25), with the 90% CI for C_max_ (0.75–0.99) slightly below the threshold. Although the AUC_0-t_ was within the acceptable range, the marginal deviation of C_max_ suggested that the rate of absorption may be slightly lower in test (S-1). In the case of olmesartan medoxomil, the AUC_0-t_ GMR was 1.08 (90% CI: 0.96–1.21), demonstrating bioequivalence in terms of extent of absorption. However, the C_max_ GMR of 1.38 (90% CI: 1.13–1.64) exceeded the upper limit of the bioequivalence criteria, indicating a significantly higher peak plasma concentration in the test (S-1) compared to that in the reference (Sevikar HCT^®^). This suggests faster or more pronounced initial absorption, which may be attributable to formulation differences [36]. For S-amlodipine besylate, both AUC_0-t_ and C_max_ GMRs (0.87 and 0.89, respectively) along with their 90% CIs were within the accepted bioequivalence range, confirming comparable bioavailability between the test (S-1) and reference (Sevikar HCT^®^).

In the dissolution test results, the test (S-1) exhibited a faster release profile of olmesartan medoxomil compared to the reference (Sevikar HCT^®^) at both pH 1.2 and pH 6.8. Correspondingly, a pharmacokinetic study in beagle dogs revealed a significantly higher C_max_ for olmesartan medoxomil (S-1). Olmesartan medoxomil is primarily absorbed in the gastrointestinal tract and is classified as a Biopharmaceutics Classification System (BCS) Class II drug, characterized by low solubility and high permeability [37]. Consequently, the dissolution rate often acts as the rate-limiting step for absorption [38]. This characteristic supports the feasibility of establishing a meaningful IVIVC because the in vitro dissolution profile possibly reflects the in vivo absorption behavior [39]. These findings underscore the importance of optimizing the dissolution characteristics during formulation to ensure consistent bioavailability. Considering the potential IVIVC, it is suggested that the dissolution rate of olmesartan medoxomil at both pH 1.2 and pH 6.8 should be modulated to more closely match that of the reference (Sevikar HCT^®^). Bioequivalence must be established in humans to ensure the therapeutic equivalence of orally administered drug products. Evidence from the literature supports the existence of a pharmacokinetic correlation between humans and beagle dogs, suggesting that the IVIVC data obtained from beagle models may be extrapolated to humans with appropriate considerations [40]. Beagle dogs offer a physiologically relevant gastrointestinal environment similar to that of humans, making them a valuable model for the early assessment of oral drugs with pH-dependent solubilities [41]. Olmesartan medoxomil, hydrochlorothiazide, and S-amlodipine besylate demonstrated comparable pharmacokinetic characteristics in both species, supporting the predictive value of beagle dogs in human pharmacokinetic modeling. Nevertheless, interspecies differences in drug absorption and metabolism should be carefully considered when interpreting the results [42,43,44,45]. Based on the pharmacokinetic findings in beagle dogs, it is essential to optimize the in vitro dissolution profiles of olmesartan medoxomil before human administration. In particular, the dissolution behavior of the test formulation should be adjusted to more closely match that of the reference product (Sevikar HCT^®^) at both pH 1.2 and pH 6.8 and improve IVIVC and ensure bioequivalence. In this context, the test (B-4), which demonstrated a higher *f*_2_ value of 92.5 at pH 6.8 compared to the *f*_2_ value of 51.9 for the test (S-1), demonstrated improved similarity in dissolution behavior relative to the reference (Sevikar HCT^®^). Based on this improvement in the in vitro dissolution profile, particularly at pH 6.8, a bioequivalence study was conducted in healthy human subjects using test (B-4). The pharmacokinetic profiles of hydrochlorothiazide, olmesartan medoxomil, and S-amlodipine besylate were compared between the test (B-4) and the reference (Sevikar HCT^®^). The GMRs of AUC_0-t_ and *C*_max_, along with their respective 90% CIs, were used as primary parameters to assess bioequivalence. For hydrochlorothiazide, the AUC_0-t_ and *C*_max_ GMRs were 0.99 and 0.97, respectively, with 90% CIs of 0.95–1.04 and 0.90–1.05. These values were well within the predefined bioequivalence range of 0.80–1.25, indicating comparable extent and rate of absorption between the two formulations. In addition, olmesartan medoxomil met the bioequivalence criteria, with an AUC_0-t_ GMR of 1.00 (90% CI: 0.94–1.05) and a *C*_max_ GMR of 0.98 (90% CI: 0.92–1.05), suggesting similar systemic exposure and peak plasma levels between the test (B-4) and reference (Sevikar HCT^®^). For S-amlodipine besylate, the AUC_0-t_ and *C*_max_ GMRs were slightly lower at 0.90 and 0.92, respectively. However, their corresponding 90% CIs (0.86–0.93 for AUC_0-t_ and 0.89–0.96 for *C*_max_) remained within the acceptable range. Collectively, these results demonstrate that the test (B-4) is bioequivalent to the reference (Sevikar HCT^®^) in terms of both the rate and extent of absorption, fulfilling the regulatory requirements for bioequivalence in human subjects.

Beyond the formulation and pharmacokinetic findings, it is also important to consider the clinical rationale for substituting racemic amlodipine with S-amlodipine in FDCs. A substantial body of clinical evidence indicates that S-amlodipine provides comparable antihypertensive efficacy to the racemate at approximately half the dose, while significantly reducing the incidence of dose-dependent adverse effects such as peripheral edema [21,22,34]. This improved tolerability is attributed to the absence of the pharmacologically inactive R(+)-enantiomer, which has been implicated in adverse vascular responses. By lowering the risk of edema, S-amlodipine-based regimens may improve long-term adherence and overall treatment outcomes in chronic hypertension management [20]. Thus, the present formulation not only demonstrates stability and bioequivalence but also carries additional clinical value, consistent with the broader chiral-switching strategy aimed at enhancing both efficacy and safety.

## 5. Conclusions

This study demonstrated that replacing amlodipine besylate with S-amlodipine besylate, its active enantiomer, in the FDC antihypertensive formulation Sevikar HCT^®^ necessitates a bilayer tablet design to ensure product stability. Single-layer tablets containing S-amlodipine besylate exhibited increased levels of unknown impurities under stress stability conditions, indicating potential interactions among active ingredients that compromise stability. The bilayer tablets effectively mitigated these stability issues by formulating S-amlodipine besylate in a separate layer. Moreover, pharmacokinetic evaluations confirmed that the bilayer formulation achieved bioequivalence with Sevikar HCT^®^, maintaining comparable efficacy. Therefore, the bilayer tablet approach is a suitable manufacturing method for developing stable and effective FDCs of antihypertensive products containing S-amlodipine besylate.

## Figures and Tables

**Table 1:** Composition of single-layer, fixed-dose combination product formulas (unit: mg).

Ingredient	Formulation Code		
	S-1	S-2	S-3
Olmesartan medoxomil	40.00	40.00	40.00
Hydrochlorothiazide	12.50	12.50	12.50
S-amlodipine besylate dihydrate	7.38	-	-
Amlodipine besylate	-	6.94	-
Pregelatinized starch	105.00	105.00	105.00
Silicified microcrystalline cellulose	118.82	112.41	112.41
Croscarmellose sodium	15.00	15.00	15.00
Magnesium stearate	1.20	1.20	1.20
Opadty II pink(85F25437)	10.00	10.00	10.00
Total weight	309.90	303.05	295.11

**Table 2:** Composition of bilayer, fixed-dose combination product formulas (unit: mg, olmesartan medoxomil [first layer], hydrochlorothiazede/S-amlodipine besylate [second layer]).

Ingredient	Formulation Code	
	B-1	B-2
Olmesartan medoxomil	40.00	40.00
Silicified microcrystalline cellulose	59.50	59.50
Pregelatinized starch	52.50	52.50
Croscarmellose sodium	15.00	15.00
Magnesium stearate	3.00	3.00
First weight	170.00	170.00
Hydrochlorothiazide	12.50	12.50
S-amlodipine besylate dihydrate	7.38	7.38
Silicified microcrystalline cellulose	39.10	39.10
Pregelatinized starch	33.02	28.27
Croscarmellose sodium	-	4.75
Magnesium stearate	3.00	3.00
Second weight	95.00	95.00
Total weight	265.00	265.00

**Table 3:** Composition of bi-layer fixed-dose combination product formulas. (unit: mg, olmesartan medoxomil, hydrochlorothiazide [first layer], S-amlodipine besylate [second layer]).

	Ingredient	Formulation Code	
		B-3	B-4
Olmesartan granule	Olmesartan medoxomil	40.00	40.00
	Silicified microcrystalline cellulose	59.50	59.50
	Pregelatinized starch	52.50	52.50
	Croscarmellose sodium	15.00	15.00
Hydrochlorothiazide powder	Hydrochlorothiazide	12.50	12.50
	Silicified microcrystalline cellulose	12.25	12.25
	Pregelatinized starch	8.15	8.15
	Silicon dioxide	2.10	2.10
	Magnesium stearate	3.00	3.00
	First weight	205.00	205.00
S-amlodipine powder	S-amlodipine besylate dihydrate	7.38	7.38
	Silicified microcrystalline cellulose	39.67	63.42
	Pregelatinized starch	20.00	53.76
	Low-substituted hydroxypropyl cellulose	25.00	15.00
	Crospovidone	-	7.50
	Silicon dioxide	0.95	0.94
	Magnesium stearate	2.00	2.00
	Second weight	95.00	150.00
	Total weight	300.00	355.00

**Table 4:** Slope *k* of the increase in total impurities in each formulation.

Formulation	Slope *k* (%/week)	R^2^
Sevikar HCT^®^	0.669	0.949
S-1	0.757	0.926
S-2	0.177	0.780
S-3	0.152	0.941
B-4	0.366	0.924

**Table 5:** Pharmacokinetic parameters after a single oral administration of the S-1 (test) and Sevikar HCT^®^ (reference) in beagle dogs.

Parameters	Hydrochlorothiazide		Olmesartan Medoxomil		S-Amlodipine Besylate	
	Test	Reference	Test	Reference	Test	Reference
AUC_0-t_ (ng/mL)	1902.0 ±378.8	2227.2 ±617.7	695.3 ±216.5	639.6 ±210.1	369.1 ±85.7	422.3 ±87.6
C_max_ (ng/mL)	1131.5 ±318.5	1320.3 ±363.0	367.5 ±108.3	266.3 ±78.7	26.5 ±6.5	29.7 ±8.1
*T*_max_ (h)	0.67 ±0.24	0.67 ±0.24	0.67 ±0.29	0.55 ±0.30	3.60 ±1.72	3.0 ±1.2
*T*_1/2_ (h)	4.83 ±2.81	4.17 ±1.27	4.21 ±1.90	4.12 ±4.11	10.91 ±3.07	10.71 ±2.63

**Table 6:** Pharmacokinetic parameters and geometric mean ratios (test/reference) with 90% confidence intervals from the bioequivalence study of S-1 (test) and Sevikar HCT^®^ (reference) in beagle dogs.

Parameters	Hydrochlorothiazide		Olmesartan Medoxomil		S-Amlodipine Besylate	
	T/R Ratio	90% CI	T/R Ratio	90% CI	T/R Ratio	90% CI
AUC_0-t_	0.85	0.81, 0.92	1.08	0.96, 1.21	0.87	0.84, 0.90
C_max_	0.86	0.75, 0.99	1.38	1.13, 1.64	0.89	0.85, 0.95

**Table 7:** Pharmacokinetic parameters after a single oral administration of B-4 (test) and Sevikar HCT^®^ (reference) in humans.

Parameters	Hydrochlorothiazide		Olmesartan Medoxomil		S-Amlodipine Besylate	
	Test	Reference	Test	Reference	Test	Reference
AUC_0-t_ (ng/mL)	524.29 ±150.48	525.16 ±129.75	6825.01 ±1929.15	6913.78 ±2105.56	125.49 ±34.10	140.81 ±41.32
C_max_ (ng/mL)	87.59 ±26.69	89.42 ±23.74	963.13 ±294.25	987.24 ±300.80	3.99 ±1.08	4.32 ±1.20
T_max_ (h)	1.5	1.5	2.5	2.0	5.0	5.0
T_1/2_ (h)	9.30 ±1.50	9.42 ±1.30	7.94 ±1.72	8.01 ±1.74	39.92 ±8.58	40.31 ±7.20

**Table 8:** Pharmacokinetic parameters and geometric mean ratios (test/reference) with 90% confidence intervals from the bioequivalence study of B-4 (test) and Sevikar HCT^®^ (reference) in humans.

Parameters	Hydrochlorothiazide		Olmesartan Medoxomil		S-Amlodipine Besylate	
	T/R Ratio	90% CI	T/R Ratio	90% CI	T/R Ratio	90% CI
AUC_0-t_	0.99	0.95, 1.04	1.00	0.94, 1.05	0.90	0.86, 0.93
C_max_	0.97	0.90, 1.05	0.98	0.92, 1.05	0.92	0.89, 0.96

## Data Availability

The data presented in this study are available upon request from the corresponding author.

## Appendix A

**Table A1:** Summary of content uniformity, assay, dissolution, and impurity test conditions for the bi-layer tablet (B-4).

Test Items	Specifications		Test Results
Content uniformity	Olmesartan medoxomil	NMT 15%	3.2%
	S-amlodipine besylate	NMT 15%	9.8%
	Hydrochlorothiazide	NMT 15%	0.7%
Assay	Olmesartan medoxomil	90.0 ~ 110.0%	102.8%
	S-amlodipine besylate	90.0 ~ 110.0%	98.2%
	Hydrochlorothiazide	90.0 ~ 110.0%	102.7%
Dissolution test	Olmesartan medoxomil	≥75% (45 min)	85%
	S-amlodipine besylate	≥70% (45 min)	83%
	Hydrochlorothiazide	≥80% (30 min)	93%
Impurity test	Amlodipine related compound A	≤0.5%	0.2%
	RNH-6270	≤2.5%	0.2%
	RNH-8276	≤0.5%	0.03%
	RNH-6373	≤0.5%	ND
	Benzothiadiazine related compound A	≤1.0%	0.04%
	Unknown impurity	≤0.2%	0.1%
	Total impurity	≤5.0%	0.9%

**Table A2:** Compatibility study of s-amlodipine besylate in stressed conditions.

		Amlodipine Related Compound A	Unknown Impurity	Total Impurity
S-amlodipine besylate	Initial	ND	ND	ND
	1w	<LOQ	<LOQ	<LOQ
	2w	<LOQ	<LOQ	<LOQ
	4w	0.06	0.16	0.30
S-amlodipine besylate + Silicified microcrystalline Cellulose	Initial	ND	ND	ND
	1w	ND	<LOQ	<LOQ
	2w	ND	<LOQ	<LOQ
	4w	<LOQ	0.21	0.31
S-amlodipine besylate + Manitol	Initial	ND	ND	ND
	1w	<LOQ	<LOQ	<LOQ
	2w	0.09	0.25	0.45
	4w	0.23	0.77	1.53
S-amlodipine besylate + Lactose	Initial	ND	ND	ND
	1w	<LOQ	<LOQ	<LOQ
	2w	<LOQ	<LOQ	<LOQ
	4w	<LOQ	0.24	0.34
S-amlodipine besylate + Pregelatinized starch	Initial	ND	ND	ND
	1w	<LOQ	<LOQ	<LOQ
	2w	<LOQ	<LOQ	<LOQ
	4w	0.05	0.22	0.39
S-amlodipine besylate + Low-substituted Hydroxypropyl cellulose	Initial	ND	ND	ND
	1w	0.06	<LOQ	0.06
	2w	0.07	0.15	0.28
	4w	0.09	0.24	0.43
S-amlodipine besylate + Croscarmellose sodium	Initial	ND	ND	ND
	1w	<LOQ	0.07	0.07
	2w	0.05	0.20	0.42
	4w	0.21	0.26	0.81
S-amlodipine besylate + Crospovidone	Initial	ND	ND	ND
	1w	<LOQ	<LOQ	<LOQ
	2w	<LOQ	0.15	0.26
	4w	0.15	0.24	0.39
S-amlodipine besylate + Sodium starch glycolate	Initial	ND	ND	ND
	1w	<LOQ	<LOQ	<LOQ
	2w	< LOQ	0.19	0.36
	4w	0.09	0.23	0.65
S-amlodipine besylate + Povidone	Initial	ND	ND	ND
	1w	0.06	<LOQ	0.06
	2w	<LOQ	015	0.28
	4w	0.09	0.24	0.65
S-amlodipine besylate + Silicon dioxide	Initial	ND	ND	ND
	1w	0.07	0.06	0.06
	2w	0.09	0.19	0.27
	4w	0.21	0.23	0.49
S-amlodipine besylate + Magnesium stearate	Initial	ND	ND	ND
	1w	<LOQ	<LOQ	<LOQ
	2w	<LOQ	<LOQ	<LOQ
	4w	<LOQ	0.23	0.30

**Table A3:** Compatibility study of olmesartan medoxomil in stressed conditions.

		RNH-6270	RNH-6373	RNH-8276	Unknown Impurity	Total Impurity
Olmesartan medoxomil	Initial	ND	ND	ND	<LOQ	<LOQ
	1w	ND	ND	<LOQ	<LOQ	<LOQ
	2w	ND	ND	<LOQ	<LOQ	<LOQ
	4w	ND	ND	<LOQ	0.05	0.05
Olmesartan medoxomil + Silicified microcrystalline Cellulose	Initial	ND	ND	ND	<LOQ	<LOQ
	1w	ND	ND	<LOQ	0.05	0.05
	2w	ND	ND	<LOQ	0.06	0.06
	4w	ND	ND	<LOQ	0.06	0.11
Olmesartan medoxomil + Manitol	Initial	ND	ND	ND	<LOQ	<LOQ
	1w	ND	ND	<LOQ	<LOQ	<LOQ
	2w	ND	ND	<LOQ	0.05	0.05
	4w	ND	ND	<LOQ	0.05	0.05
Olmesartan medoxomil + Lactose	Initial	ND	ND	ND	<LOQ	<LOQ
	1w	ND	ND	<LOQ	0.05	0.05
	2w	ND	ND	<LOQ	0.05	0.05
	4w	ND	ND	<LOQ	0.05	0.10
Olmesartan medoxomil + Pregelatinized starch	Initial	ND	ND	ND	<LOQ	<LOQ
	1w	ND	ND	<LOQ	<LOQ	<LOQ
	2w	ND	ND	<LOQ	<LOQ	<LOQ
	4w	ND	ND	<LOQ	0.05	0.10
Olmesartan medoxomil + Low-substituted Hydroxypropyl cellulose	Initial	ND	ND	ND	<LOQ	<LOQ
	1w	ND	ND	<LOQ	<LOQ	<LOQ
	2w	ND	ND	<LOQ	0.05	0.05
	4w	ND	ND	<LOQ	0.05	0.10
Olmesartan medoxomil + Croscarmellose sodium	Initial	ND	ND	ND	<LOQ	<LOQ
	1w	ND	ND	<LOQ	0.05	0.05
	2w	ND	ND	<LOQ	0.05	0.05
	4w	ND	ND	<LOQ	0.10	0.23
Olmesartan medoxomil + Crospovidone	Initial	ND	ND	ND	<LOQ	<LOQ
	1w	ND	ND	<LOQ	<LOQ	<LOQ
	2w	ND	ND	<LOQ	<LOQ	<LOQ
	4w	ND	ND	<LOQ	0.05	0.10
Olmesartan medoxomil + Sodium starch glycolate	Initial	ND	ND	ND	<LOQ	<LOQ
	1w	ND	ND	<LOQ	<LOQ	<LOQ
	2w	ND	ND	<LOQ	<LOQ	<LOQ
	4w	ND	ND	<LOQ	0.05	0.05
Olmesartan medoxomil + Povidone	Initial	ND	ND	ND	<LOQ	<LOQ
	1w	ND	ND	<LOQ	<LOQ	<LOQ
	2w	ND	ND	<LOQ	0.06	0.06
	4w	ND	ND	<LOQ	0.06	0.10
Olmesartan medoxomil + Silicon dioxide	Initial	ND	ND	ND	<LOQ	<LOQ
	1w	ND	ND	<LOQ	0.05	0.05
	2w	ND	ND	<LOQ	0.07	0.14
	4w	ND	ND	<LOQ	0.10	0.23
Olmesartan medoxomil + Magnesium stearate	Initial	ND	ND	ND	<LOQ	<LOQ
	1w	ND	ND	<LOQ	0.05	0.05
	2w	ND	ND	<LOQ	0.05	0.05
	4w	ND	ND	<LOQ	0.05	0.11

**Table A4:** Compatibility study of hydrochlorothiazide in stressed conditions.

		Benzothiadiazine Related Compound A	Unknown Impurity	Total Impurity
Hydrochlorothiazide	Initial	<LOQ	0.10	0.10
	1w	<LOQ	0.08	0.08
	2w	<LOQ	0.08	0.08
	4w	<LOQ	0.08	0.08
Hydrochlorothiazide + Silicified microcrystalline Cellulose	Initial	<LOQ	0.10	0.10
	1w	<LOQ	0.10	0.10
	2w	<LOQ	0.10	0.10
	4w	<LOQ	0.10	0.10
Hydrochlorothiazide + Manitol	Initial	<LOQ	0.09	0.09
	1w	<LOQ	0.08	0.08
	2w	<LOQ	0.07	0.07
	4w	<LOQ	0.08	0.08
Hydrochlorothiazide + Lactose	Initial	<LOQ	0.09	0.09
	1w	<LOQ	0.08	0.08
	2w	<LOQ	0.08	0.08
	4w	<LOQ	0.07	0.07
Hydrochlorothiazide + Pregelatinized starch	Initial	<LOQ	0.10	0.10
	1w	<LOQ	0.09	0.09
	2w	<LOQ	0.08	0.08
	4w	<LOQ	0.07	0.07
Hydrochlorothiazide + Low-substituted Hydroxypropyl cellulose	Initial	<LOQ	0.10	0.10
	1w	<LOQ	0.07	0.07
	2w	<LOQ	0.08	0.08
	4w	<LOQ	0.07	0.07
Hydrochlorothiazide + Croscarmellose sodium	Initial	<LOQ	0.10	0.10
	1w	<LOQ	0.08	0.08
	2w	<LOQ	0.08	0.08
	4w	<LOQ	0.07	0.07
Hydrochlorothiazide + Crospovidone	Initial	<LOQ	0.10	0.10
	1w	<LOQ	0.09	0.09
	2w	<LOQ	0.06	0.06
	4w	<LOQ	0.07	0.07
Hydrochlorothiazide + Sodium starch glycolate	Initial	<LOQ	0.09	0.09
	1w	<LOQ	0.08	0.08
	2w	<LOQ	0.07	0.07
	4w	<LOQ	0.07	0.07
Hydrochlorothiazide + Povidone	Initial	<LOQ	0.10	0.10
	1w	<LOQ	0.13	0.13
	2w	<LOQ	0.07	0.07
	4w	<LOQ	0.07	0.07
Hydrochlorothiazide + Silicon dioxide	Initial	<LOQ	0.10	0.10
	1w	<LOQ	0.09	0.09
	2w	<LOQ	0.08	0.08
	4w	<LOQ	0.08	0.08
Hydrochlorothiazide + Magnesium stearate	Initial	<LOQ	0.10	0.10
	1w	<LOQ	0.09	0.09
	2w	<LOQ	0.06	0.06
	4w	<LOQ	0.05	0.05
